# Physicochemical and antioxidant activity of fruit harvested from eight jujube (*Ziziphus jujuba Mill*.) cultivars at different development stages

**DOI:** 10.1038/s41598-022-06313-5

**Published:** 2022-02-10

**Authors:** Min Yan, Yan Wang, Ritesh Balaso Watharkar, Yunfeng Pu, Cuiyun Wu, Minjuan Lin, Dengyang Lu, Mingzhe Liu, Jingkai Bao, Yilei Xia

**Affiliations:** 1 College of Plant Sciences, Tarim University, Alar, 843300 Xinjiang China; 2 College of Life Sciences, Tarim University, Alar, 843300 Xinjiang China; 3 College of Management, Agri and Food Management, MIT-Art Design and Technology University, Loni-Kalbhor, Rajbaugh, Solapur-Pune Highway, Pune, Maharashtra 412201 India; 4 The National-Local Joint Engineering Laboratory of High Effciency and Superior-Quality Cultivation and Fruit Deep Processing Technology on Characteristic Fruit Trees, Alar, 843300 Xinjiang China; 5 Xinjiang Production and Construction Corps Key Laboratory of Protection and Utilization of Biological Resources in Tarim Basin, Alar, 843300 Xinjiang China

**Keywords:** Carbohydrates, Plant physiology, Secondary metabolism

## Abstract

Jujube is a crop highly resistant to drought and salinity, making it one of the main fruit trees in Xinjiang. The present study evaluated the changes in the physicochemical and antioxidant activities of jujube fruit of eight different cultivars from Xinjiang, China. The developmental stages were selected according to the days after full bloom and fruit peel colour during ripening; these stages included young (S1), fruit core-hardening (S2), green ripening (S3), half-red maturity (S4) and complete red. In present study, different cultivars of jujube fruit showed similar chemical profiles, but their amounts showed great variation. HZ had the highest content of sugars, and JY had the highest content of cAMP and cGMP, while relatively higher levels of ascorbic acid, catechin, epicatechin, rutin, proanthocyanidin and antioxidant activity were found in ‘FS’ than in other cultivars, indicating that ‘FS’ could be used as a potential natural antioxidant. Regarding the development stages of jujube fruit, the moisture, ascorbic acid, total polyphenol, catechin, epicatechin, proanthocyanidin and rutin contents decreased during the development of all jujube cultivars, while the fructose, glucose, sucrose, cAMP, and cGMP contents greatly increased. The antioxidant activity determined by DPPH and ABTS radical scavenging decreased as the fruits matured. Therefore, the results suggest that green jujube (S1) could be used for natural antioxidants (catechin, epicatechin, proanthocyanidin) and that the advanced ripening stage(S5) is the proper picking period for fresh fruit and commercial processing.

## Introduction

Jujube (*Ziziphus jujuba Mill.*) belongs to the *Rhamnaceae* family, and it is generally recognized as the most important *Ziziphus* species for fruit production. The plant is indigenous to China (especially in the north) and is widely distributed in the temperate and subtropical areas of the Northern Hemisphere^1^. In 2019, jujube fruit production in China was approximately 7.46 million tons^2^, which comprised 90% of the total worldwide production. Jujube fruit has an exquisite taste, unique flavor, and high nutritional value which make it popular and profitable. This fruit is commonly consumed as a food additive, and fruit flavoring agent and has been used for medicinal purposes in Asian countries for thousands of years^3^. Jujube fruit has been reported to be high in carbohydrates, organic acids, amino acids^4^, vitamin C and minerals^5^. Recent studies have reported that jujube fruit provides health benefits such as antioxidant and anticancer, hepatoprotective, gastrointestinal protective, anti-inflammatory, anti-insomnia, immunostimulating, and neuroprotective effects^6^. These medicinal properties have been shown to be closely related to the composition of jujube’s bioactive components. Some bioactive components of jujube, such as cyclic adenosine monophosphate^7^, ϒ-aminobutyric acid^8^, phenolic acids^9^, flavonoids and saponins^10^, have been investigated in previous studies.

Factors such as environment, type of cultivar, harvesting practice and storage conditions significantly affect fruit quality, and consumers consider only quality fruits. Therefore, research on the quality of horticultural crops is necessary to determine the loss of quality due to various factors so that cultivars can provide quality fruits to consumers. The fruit quality affected by the developmental stage has attracted much research attention in recent years. Some studies have reported changes in nutritional compounds, total phenols, and antioxidant activity during the development of jujube fruit^5,11–14^, others have studied the changes in nutritional compounds, sensory characteristics and volatile compounds^15–19^, while only a few researchers have studied the changes in phenolic compounds and antioxidant activity during jujube fruit development^4,20,21^. China also has approximately 944 cultivars of jujube fruit in which the physicochemical, physiological, and functional characteristics differ according to cultivar^22^. Therefore, it is necessary to learn the nutritional components, bioactive components and antioxidant properties of major domesticated jujube cultivars at various developmental stages, which would lead to a better understanding of their nutritional and medicinal effects on human health.

Most studies have been carried out on jujube cultivars grown in traditional jujube planting regions in China. Although Xinjiang is the major province for the cultivation of jujube fruit, with a recorded 445,225 ha of cultivated land and an annual production of approximately 3.72 megatons^23^, no reliable scientific reports were found regarding the effect of developmental stage on the nutritional and functional aspects of jujube fruit cultivated in Xinjiang. Therefore, the objective of the present study was to analyze the changes in the nutritional components, bioactive components and antioxidant activity of eight jujube cultivars grown in Xinjiang. The focus of the study was to facilitate and identify a suitable ripening stage and optimal harvest time to obtain fruit containing high nutritional and functional characteristics.

## Materials and methods

### Chemicals

All reagents, specifically 2,2´-diphenyl-1-picrylhydrazyl (DPPH), 2,2´-azino-bis (3-ethylbenzothiazoline-6-sulfonic acid) diammonium salt (ABTS), and 6-hydroxy-2,5,7,8-tetramethylchroman-2-carboxylic acid (Trolox), were purchased from Sigma–Aldrich (St. Louis, MO, USA). HPLC-grade phenolic standards (protocatechuic acid, (+)-catechin, p-hydroxybenzoic acid, chlorogenic acid, (−)-epicatechin, caffeic acid, p-coumaric acid, ferulic acid, rutin, quercetin, quercetin-3-rhamnoside, cAMP, and cGMP) were also purchased from Sigma–Aldrich (St. Louis, MO, USA). HPLC grade sugar standards (glucose, fructose, and sucrose), ascorbic acid (AA), phloridzin and kaempferol were purchased from Yuanye Biotechnology Ltd. (Shanghai, China). Methanol, acetonitrile and formic acid were purchased from Tedia Company, Inc. (Fairfield, CT, USA). Folin-Ciocalteu reagent and all other chemicals were obtained from Sinopharm Chemical Reagent Co. (Shanghai, China).

### Sample collection

Fruits were collected from the jujube germplasm resources nursery of Tarim University (40° 32′ 23.50″ N, 81° 17′ 41.61″ E), Alar, Xinjiang, China. In this experiment,permission for sample collection was granted by Tarim University. Jujube plants of the same age, structure, and consistent management level were selected, and five trees were sampled for each cultivar. All methods involving plants were carried out in accordance with relevant national, international, or institutional guidelines and regulations.

**Figure 1:**
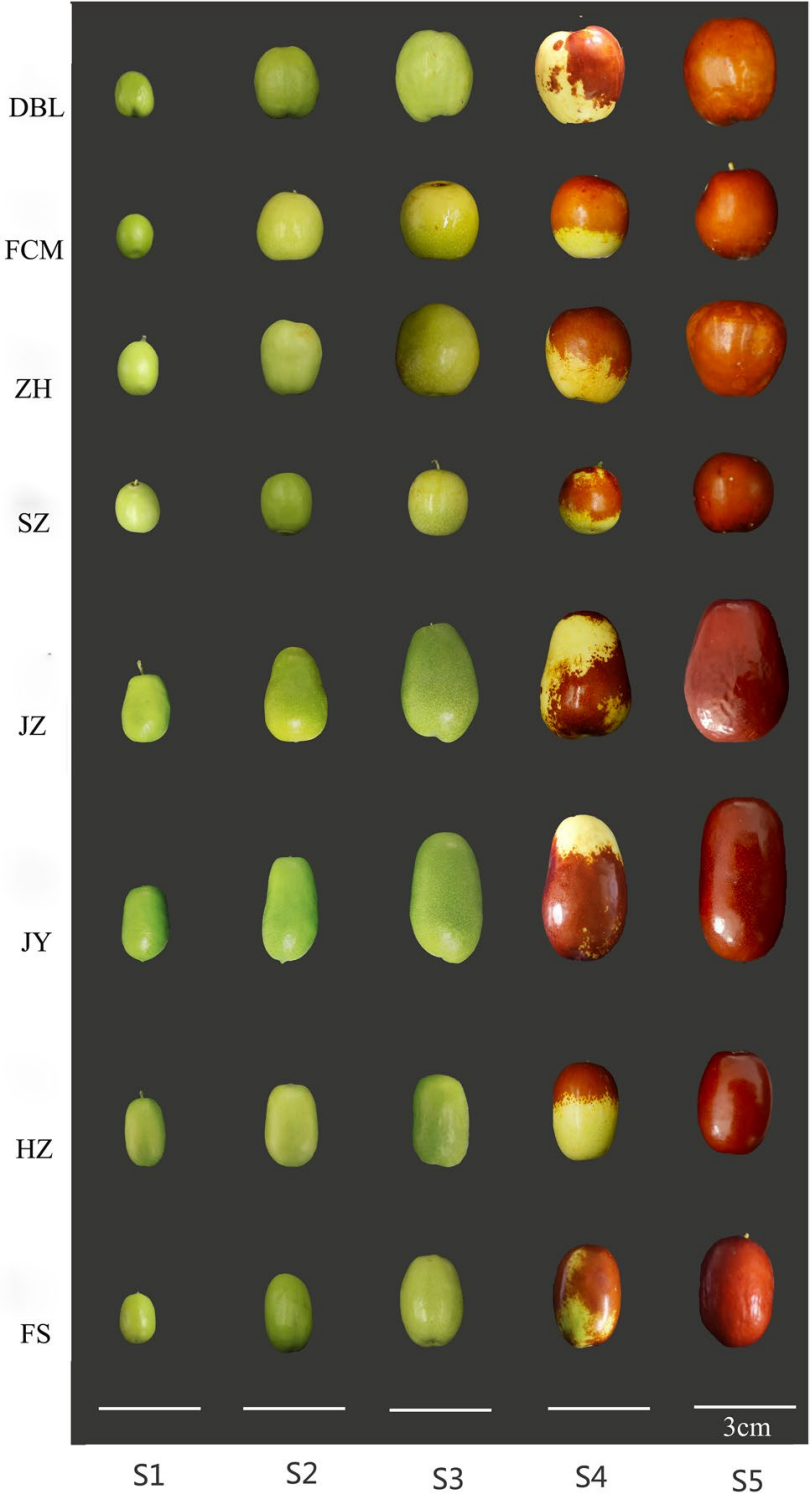
Photographs of jujube fruits used in this experiment.

A total of eight cultivars of *Z. jujuba* were investigated*,* including Dabailing (DBL), Junzao (JZ), Junyou (JY), Spinosa (SZ), Huizao (HZ), Zanhuang (ZH), Fushuai (FS), and Fucuimi (FCM). The developmental stages were selected as per the stages of jujube fruit reported by Zhang^1^. Jujube fruits were collected from July to October 2019 at five stages (Fig. 1): the young fruit stage (S1); the fruit core-hardening stage (S2); the green ripe stage, in which the peel is green (S3); the half-red maturity stage, in which 40–60% of the surface area is red (S4); and the red maturity stage, in which 100% of the surface area is red (S5). Twenty jujube fruits were picked randomly for each sample from different parts of five trees of the same species, which were free of any visible blemishes or diseases. The moisture and AA contents of the fresh fruit were measured immediately after the fruit had been transported to the laboratory. The sliced fruit was then frozen using a freeze dryer (The FreeZone 6, Labconco, USA). Lyophilized samples were homogenized in a domestic blender (JYL-C022E, Jiuyang, China), and the powder was packed in airtight polyethylene bags. Finally, the powder was stored at − 80 °C before further analysis.

### Determination of moisture content and titratable acid (TA)

The moisture content and titratable acid (TA) were measured according to the method described by Pu^24^, and the TA results were expressed in terms of % malic acid.

### Determination of ascorbic acid (AA)

The ascorbic acid content was determined according to the method described by Wojdyło^25^ with some modifications. In this analysis, 2 to 3 g of sliced fresh fruit was used. The sample was added to 50 mL of 2% oxalic acid solution and homogenized for 10 min using a magnetic stirrer. The homogenized suspension was centrifuged at 3,000 rpm for 15 min, and the supernatant was collected and filtered with a 0.22 μm syringe filter for chromatographic analysis. High-performance liquid chromatography was performed using an LC-20A system (Shimadzu, Kyoto, Japan) equipped with two LC-20AB pumps, an SIL-20A injector, and an SPD-M20A detector. A C18 column (I.D. 250 × 4.6 mm; Dikma Ltd, Tianjin, China) was used to separate the compounds. A 10 μL aliquot of the sample was injected into the system where the column oven temperature was adjusted to 30 °C. The samples were eluted using 0.5% formic acid at a flow rate of 0.6 mL/min. Absorbance was monitored at 243 nm, where L-ascorbic acid was used as the standard. The AA concentration was expressed as g/kg FW.

### Determination of fructose, glucose and sucrose

Sugar profiles of jujube fruit were produced using HPLC, as per the method described by Pu^24^, and the results were expressed as g/kg DW.

### Determination of cyclic adenosine monophosphate (cAMP) and cyclic guanosine monophosphate (cGMP)

The method described by Chen^26^ was slightly modified to measure cAMP and cGMP. Briefly, 5 g of sample was mixed with 100 mL of deionized water in a volumetric flask. The flask was placed in an ultrasonic bath for 30 min. Then, the extract was centrifuged (3000 rpm for 15 min), and the collected supernatant was filtered using a 0.22 μm syringe filter. The chromatographic system (Shimadzu, Kyoto, Japan) consisted of an LC-20A system equipped with two pumps (LC-20AB), an injector (SIL-20A), and a detector (SPD-M20A). The compounds were separated using a C18 column (I.D. 250 × 4.6 mm, Dikma Ltd, Tianjin, China) at 30 °C. The filtered extract (aliquot) was used for injection, while methanol/water/formic acid (89.5:10: 0.5, v/v/v) was used as the mobile phase, which was pumped at a flow rate of 0.7 mL min^−1^. The absorbance was monitored at 256 nm.

### Determination of total polyphenol content (TPC) and total flavonoid content (TFC)

The total polyphenol content (TPC) was determined according to the Folin–Ciocalteu colorimetric method^24^, and the results were expressed as gallic acid equivalents per gram of fruit (g GAE/kg DW).

The total flavonoid content (TFC) was evaluated as per the process described by Pu^24^, and the results were expressed as milligrams of rutin equivalent per gram of fruit (g RE/kg DW).

### Determination of phenolic profiling

The phenolic profile was produced according to the method described previously by Pu^24^, with some modification. HPLC analysis was performed on a Shimadzu (Kyoto, Japan) LC-20A system equipped with two pumps (LC-20AB), an injector (SIL-20A), and a detector (SPD-M20A). Separation was performed on a ZORBAX SB-C18 column (I.D. 250 × 4.6 mm, Agilent, USA). The column oven temperature was set to 30 °C. The absorbance was monitored at 253, 279, 284, 310, 324, 327, 355, and 370 nm. The solvent system had a constant flow rate of 0.7 mL/min and a 10 μL injection volume. Solvents such as 0.5% formic acid (eluant B) and methanol (eluant A) were used for the mobile phase. The gradient programs for the samples were as follows: 0–6 min, 90% B; 6–10 min, 90–80% B; 10–11 min, 80–75% B; 11–15 min, 75–70% B; 15–25 min, 70–60% B; 25–32 min, 60–45% B; 32–40 min, 45–0% B; 40–50 min, 0% B; 50–51 min, 0–90% B; 51–60 min, 90% B. The injection volume was maintained at 10 μL, and the constant temperature remained at approximately 30 °C. Detection wavelengths were chosen based on the absorption maximums of the UV spectra of the selected phenolic compounds.

### Antioxidant activity assay

A DPPH radical scavenging assay was performed according to the method adopted by Tian^27^ with some modifications. A 20 µL aliquot of the extract was mixed with 160 µL DPPH solution in a 96-well microplate. The mixture was vortexed at 300 rpm in a dark environment for 30 min, and the absorbance was measured using a plate reader (1510, Thermo Fisher, USA) at 517 nm. Trolox was used as a standard, and the results were expressed in mmol TE/kg DW.

The ABTS radical assay was performed according to the method adopted by Oney-Montalvo^28^ with some modifications. A 20 µL aliquot of the extract was mixed with 170 µL ABTS solution in a 96-well microplate. The mixture was vortexed at 300 rpm in the dark for 10 min, and the absorbance was measured using a plate reader (1510, Thermo Fisher, USA) at 734 nm. Trolox was used as a standard, and the results were expressed as mmol TE/kg DW.

### Statistical analysis

SPSS version 20 (SPSS Inc., Chicago, IL, USA) was used to perform statistical analysis of the collected data. The data were analyzed using one-way analysis of variance (ANOVA), and Duncan’s test was performed to determine correlations at the p < 0.05 significance level. Furthermore, principal composition analysis (PCA) was performed using Unscrambler 10.1 (CAMO AS, Trondheim, Norway, https://www.aspentech.com/en/acquisition/camo-analytics) software.

## Results and discussion

### Change in moisture content

**Figure 2:**
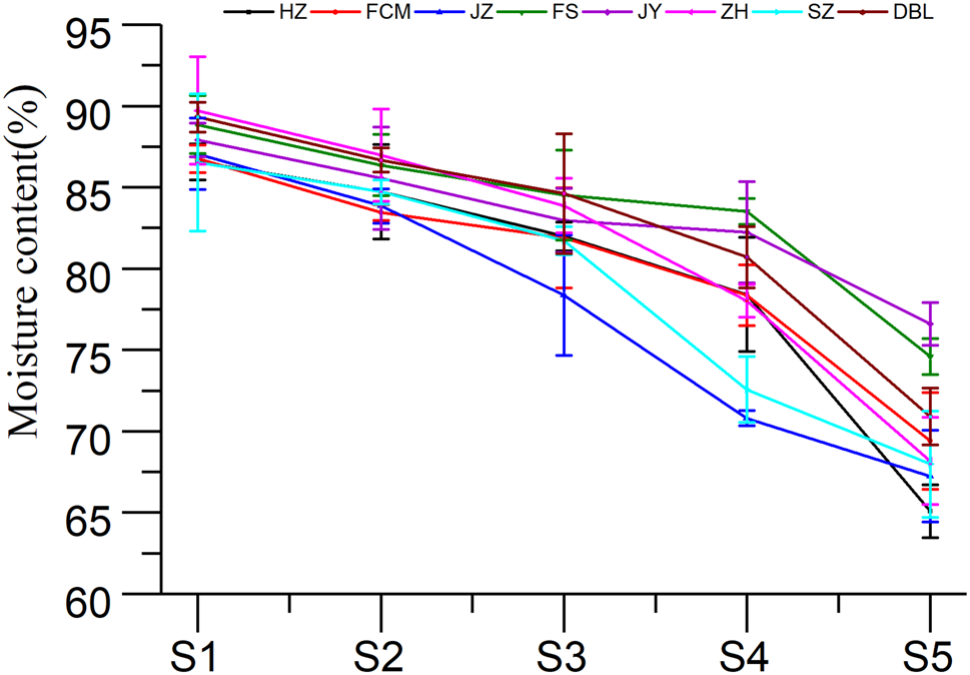
Change in moisture content of jujube fruit at various developmental stages.

Moisture content has been considered a critical parameter to evaluate the quality of jujube fruits, and it can be significantly affected by genotype and cultivation conditions^26^. The change in moisture content in the eight jujube cultivars at different developmental stages is shown in Fig. 2. A significant change was observed in all cultivar samples at various developmental stages. In this study, the highest moisture content varied between 89.31% (DBL) and 86.52% (SZ) in the young fruit stage (S1), whereas the lowest varied between 65.09% (HZ) and 76.60% (JY) in the red maturity stage (S5). Overall, the moisture content was found to decrease from the young stage (S1) to the red maturity stage (S5) in all cultivars. The decrease in moisture during the maturation process may be due to water evaporation from the fruit, which led to a further concentration of dry matter^29^. The inconsistent trend in the results may be due to climate and temperature changes at twilight during the different maturity stages, resulting in the accumulation of solids in the fruit.

### Change in titratable acid (TA)

**Figure 3:**
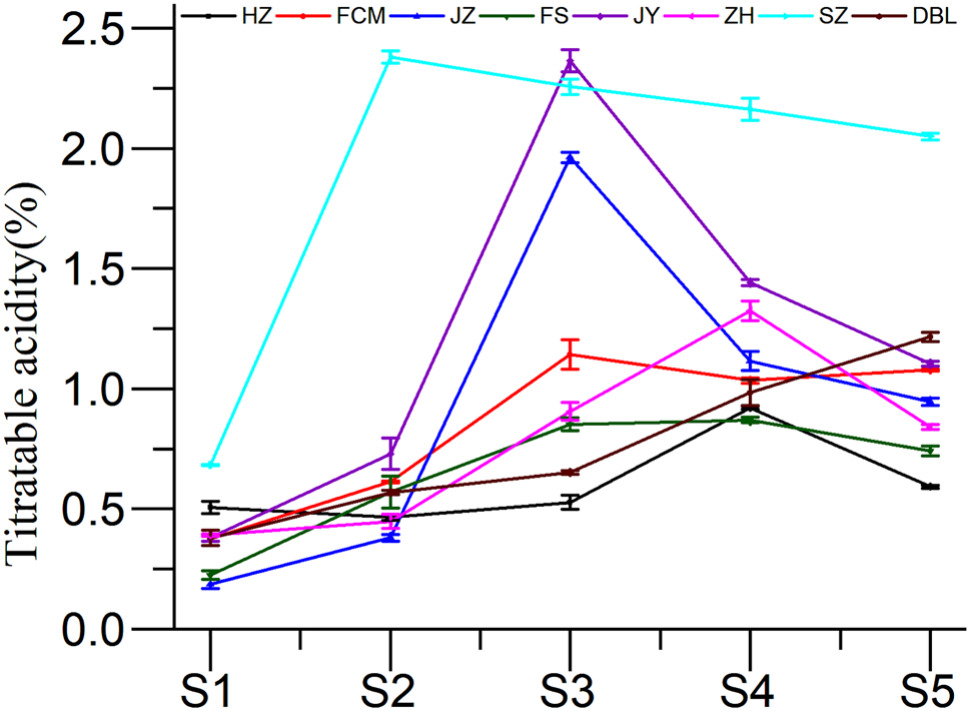
Change in titratable acid content of jujube fruit at various developmental stages.

Titratable acidity describes the accumulated acid in the fruits during development, and it tends to decrease as the fruits mature^26^. In the present investigation, the TA of all jujube fruit cultivars at different developmental stages showed significant differences at the *P* < 0.05 level (Fig. 3). The TA content of DBL was found to be increased with respect to fruit maturity, whereas FCM showed an increasing trend up to the S3 stage and remained constant thereafter. In addition, the TA in other cultivars (HZ, JZ, FS, JY, ZH, and SZ) increased during the advanced development stage (up to S3) and decreased gradually thereafter. These findings are consistent with previous studies by Wu^12^. Among all cultivars, SZ showed the highest concentration of TA, which was observed to almost double at the final stage of development. The fluctuation in concentration during developmental stages may be due to a similar trend in ethylene production^30^.

### Change in ascorbic acid

**Figure 4:**
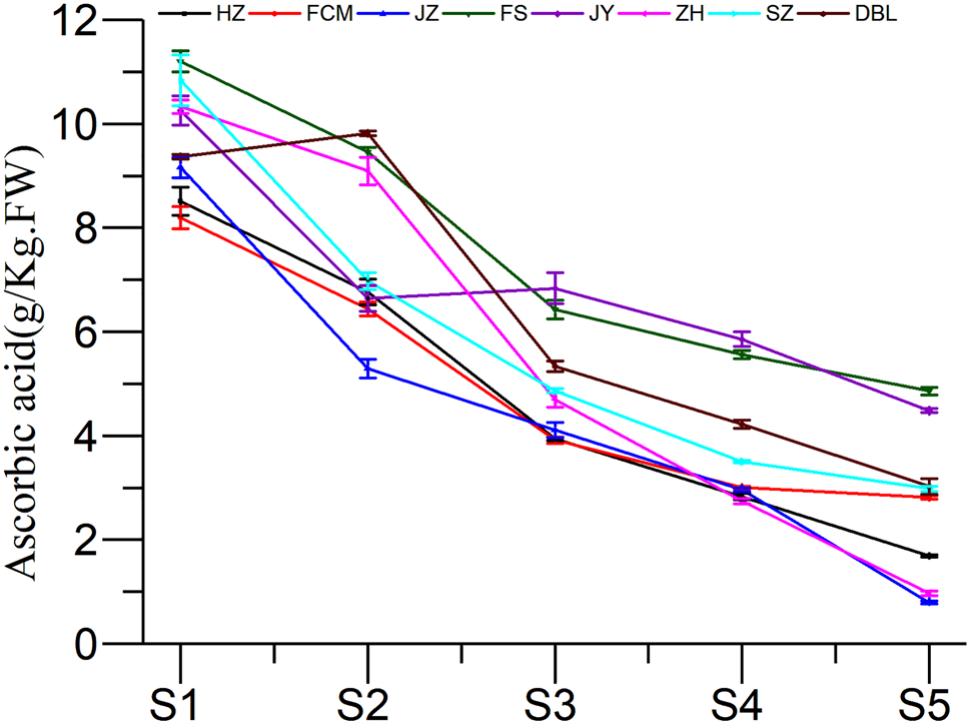
Change in ascorbic acid content of jujube fruit at various developmental stages.

Ascorbic acid is a water soluble compound mainly known for its antioxidant properties^16^, and it has been reported to scavenge free radicals by inhibiting radical chain reactions^17^. This compound also acts as a reducing and chelating agent^14^. Jujube fruit can be considered a good source of ascorbic acid in the diet, and, on average, a 20 g portion of jujube fruit can meet an adult’s daily requirement for ascorbic acid^31^. In this study, the AA concentration was found to be significantly different (*P* < 0.05) at different developmental stages in all cultivars (Fig. 4). The highest AA concentrations varied between 8.51 g/kg FW (HZ) and 11.20 g/kg FW (FS) in the young fruit stage (S1), whereas the lowest varied between 0.79 g/kg FW (JZ) and 4.86 g/kg FW (FS) in the red maturity stage (S5). Overall, the AA content was found to decrease from the young stage (S1) to the red maturity stage (S5) in all cultivars. Wu^12^ reported similar findings in the ripening stages of ‘pear-jujube’ (*Zizyphus jujube* Mill.). FS might be considered a potential commercial cultivar due to its having the highest amount of AA compared to other commercial cultivars at the young stage (S1) and fully mature stage (S5). The reason behind all this variation in the accumulation of ascorbic acid may mainly be due to fruit genotype, cultivation condition, and management application^26^.

### Change in fructose, glucose and sucrose

**Figure 5:**
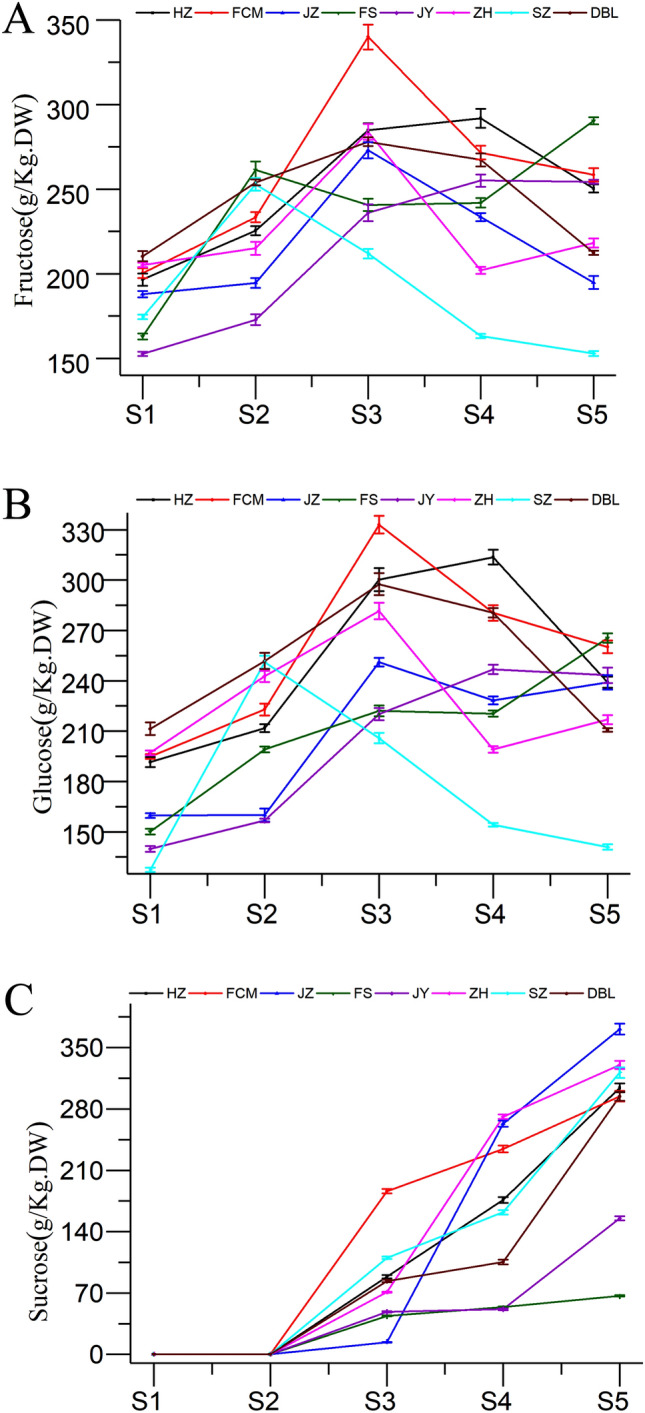
Changes in (**A**) fructose, (**B**) glucose, and (**C**) sucrose contents of jujube fruit at various developmental stages.

The sugar content in fruits helps to increase the sweet taste and aroma necessary to maximize consumer acceptance^26,32^. In this study, fructose, glucose, and sucrose were determined at different maturity stages and are presented in Fig. 5. As shown in Fig. 5C, sucrose was initially undetected in early developmental stages (S1 and S2) but started to appear thereafter; then, the levels substantially increased, and sucrose became the dominant sugar in most of the jujube cultivars (Fig. 5C). Glucose and fructose exhibited an increasing tendency during the pre-aging period and decreased thereafter (Fig. 5A,B). This finding was similar to those of studies reported by Wu^12^ and Song^14^. The decreases in fructose and glucose may be due to the reduction of sugars to polysaccharides and participation in various physical and chemical reactions^12^. In addition, the fruit weight increased by between 54 and 112% from stages S3 to S5, indicating that the concentration of fructose and glucose may have become diluted as development progressed.

Interestingly, the highest amounts of fructose, glucose and sucrose were FS (290.38 g/kg DW), FS (265.49 g/kg DW) and JZ (371.31 g/kg DW) respectively in the red maturity stage (S5), whereas the highest amount of total sugar was recorded in the HZ cultivar (813.26 g/kg DW), followed by the JZ cultivar (805.28 g/kg DW). Combined with their low moisture content in the fully red stage (S5), it seems to make sense of the situation that JZ and HZ are the main cultivars planted for dried fruit processing in Xinjiang. In conclusion, the sucrose and total sugar contents of jujube fruit showed an increasing tendency during ripening. These results corresponded with the fact that the sweetness of jujube fruits rapidly increases after the green ripening stage (S3), confirming that the accumulation of sugars in jujube fruits occurs mainly in the later stages of development^33^.

### Change in cAMP and cGMP

**Figure 6:**
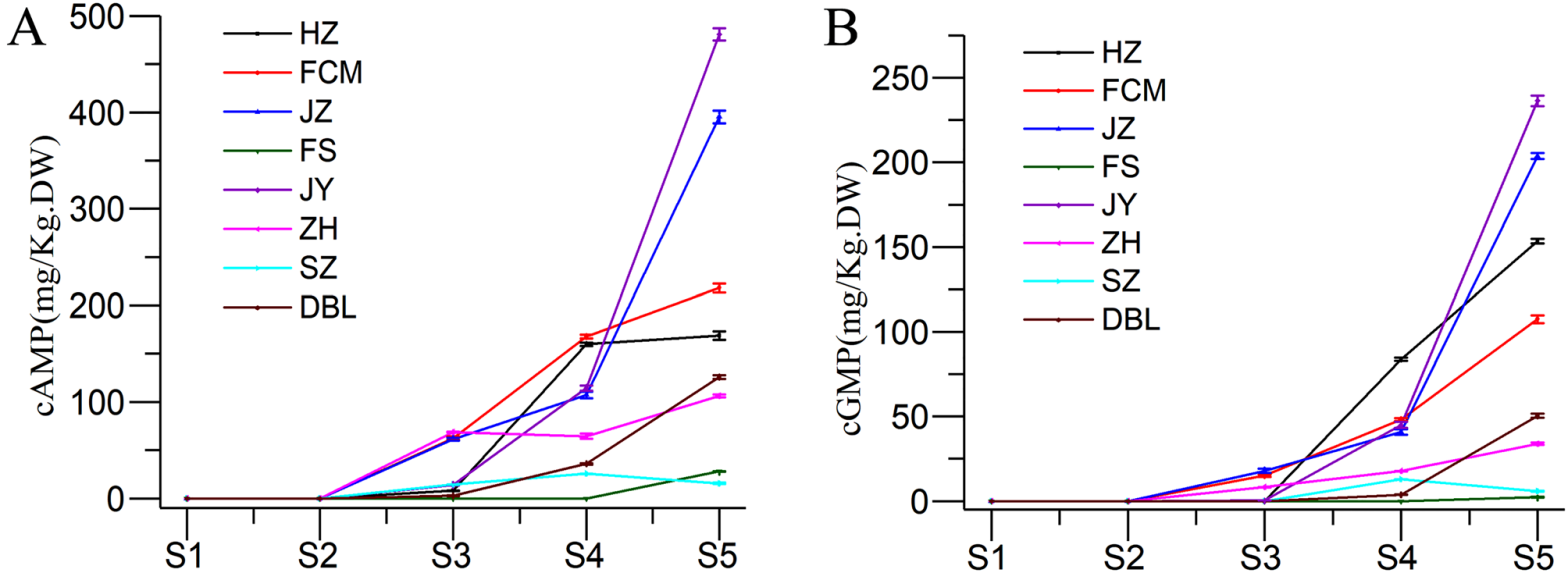
Changes in (**A**) cAMP and (**B**) cGMP of jujube fruit at various developmental stages.

Cyclic adenosine monophosphate (cAMP) and cyclic guanosine monophosphate (cGMP) are significant secondary messengers in plants and are related to cell growth and differentiation, and they also have anti-inflammatory effects^7^. The obtained cAMP content of jujube fruit ranged from 30 to 160 mg/kg, which is the highest value observed in more than 180 plants^7^. In the present study, significant differences were found in the cAMP and cGMP concentrations of jujube fruit (Fig. 6). Similar to sucrose, cAMP and cGMP were initially undetected in early developmental stages (S1 and S2, Fig. 6A,B) but started to appear thereafter; then, the levels substantially increased and reached the highest amounts in the fully ripened stage (S5). At the fully ripened stage (S5), the JY cultivar contained the highest amount of cAMP (480.92 mg/kg DW) and cGMP (236.39 mg/kg DW), followed by JZ, FCM, HZ, DBL, ZH, FS and SZ. The SZ cultivar had the lowest level of cAMP (15.51 mg/kg DW) and cGMP (5.91 mg/kg DW). As per the results obtained in this study, the cultivar is the major factor affecting cAMP and cGMP accumulation in jujube fruit. Moreover, the contents of cAMP and cGMP might be potential indices for selecting the proper picking time.

### Change in total phenolic content (TPC)

**Figure 7:**
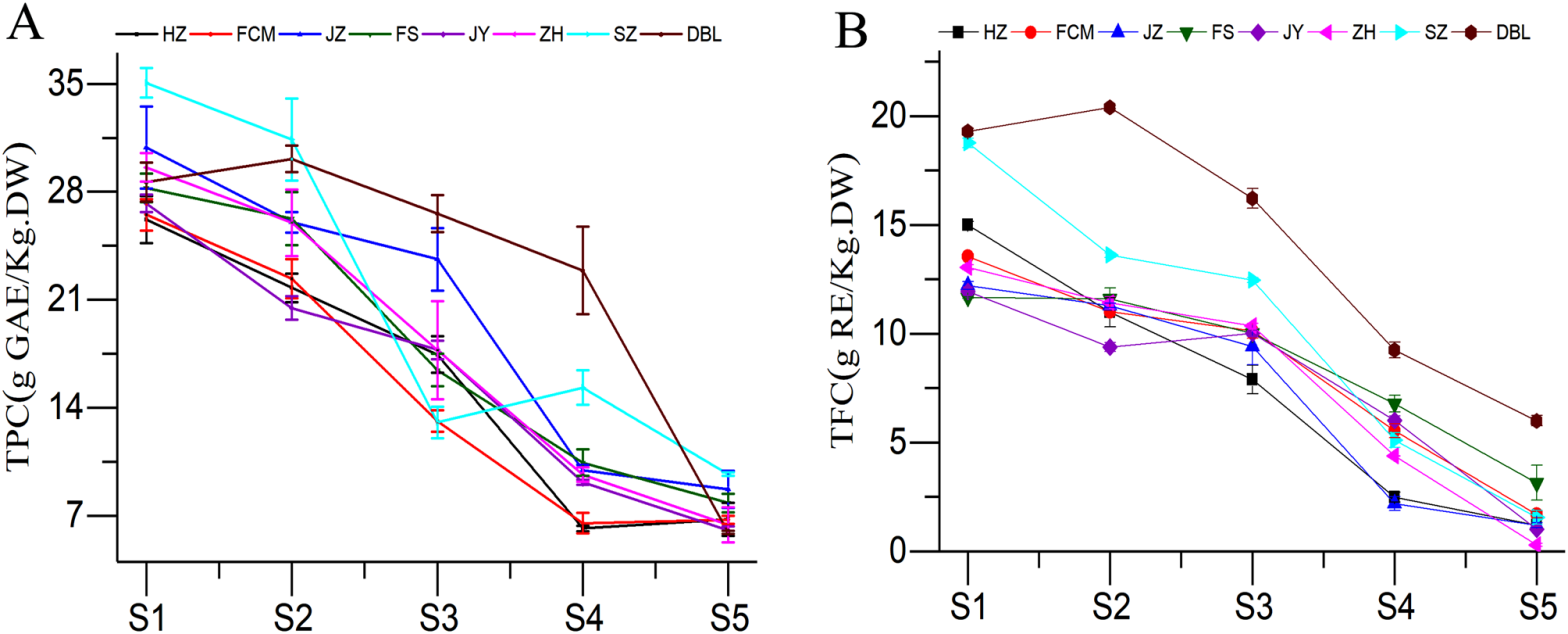
Changes in (**A**) total phenol content (TPC) and (**B**) total flavonoid content (TFC) of jujube fruit at various developmental stages.

Jujube fruit is a promising source of antioxidants due to the considerable amount of phenolic compounds. Significant differences (*P* < 0.05) were determined in the TPC concentration t in the various developmental stages (Fig. 7A). Generally, jujube fruit from the eight cultivars exhibited similar total phenolic patterns, although a decreasing trend was observed as the fruit developed. Among all cultivars, the highest TPC was found in the SZ cultivar (35.06 g GAE/kg), followed by HZ (26.19 g GAE/kg DW), whereas the lowest TPC was found at the red maturity stage (S5) in the DBL cultivar (5.92 g GAE/kg DW), followed by SZ (9.68 g GAE/kg DW). The TPC decreased rapidly up to the S3 stage and thereafter decreased slowly in all the cultivars. The decreasing trend in TPC was consistent with previously reported studies^11,21^. Moreover, the TPC concentration was found to be higher in the S5 stage than in studies reported by Wang^11^ and Wang^21^. These differences may be due to the different cultivars, tissue structures, harvesting times, climatic conditions, and study locations at high altitudes^34^.

### Change in total flavonoid content (TFC)

Flavonoids act as secondary metabolites that help to provide nutritional and sensory attributes to fruit trees and possess multiple health-promoting properties^26^. Flavonoids are an essential component for various applications as nutraceuticals, pharmaceuticals and medicinal foods due to their antioxidative, anti-inflammatory, antimutagenic, and anticarcinogenic properties^35^. The TFC was found to be significantly different (*P* < 0.05) in all developmental stages (Fig. 7B). In this study, a decreasing trend in TFC was found as all cultivars ripened. The maximum TFC was recorded in the DBL cultivar (19.30 g RE/kg DW) in the young stage (S1), whereas the lowest was recorded in the ZH cultivar (0.29 g RE/kg DW) in the red maturity stage (S5). Wang^11,21^ reported a similar trend in terms of total flavonoids present in jujube fruit. The present findings clearly indicate that the TPC concentration varies according to maturity and cultivar, which indicates that different districts with different climate conditions might experience different stresses on the synthesis of flavonoids during jujube fruit development^26^.

### Phenolic compounds profiling

**Figure 8:**
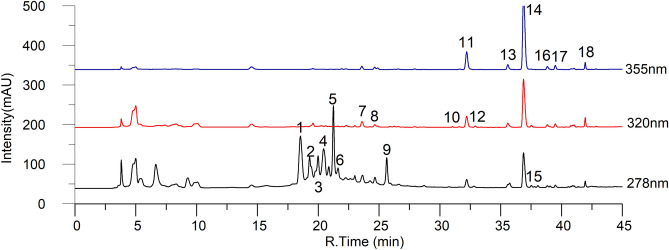
The HPLC chromatograph of phenolic profiles (FS at stage 1).

**Figure 9:**
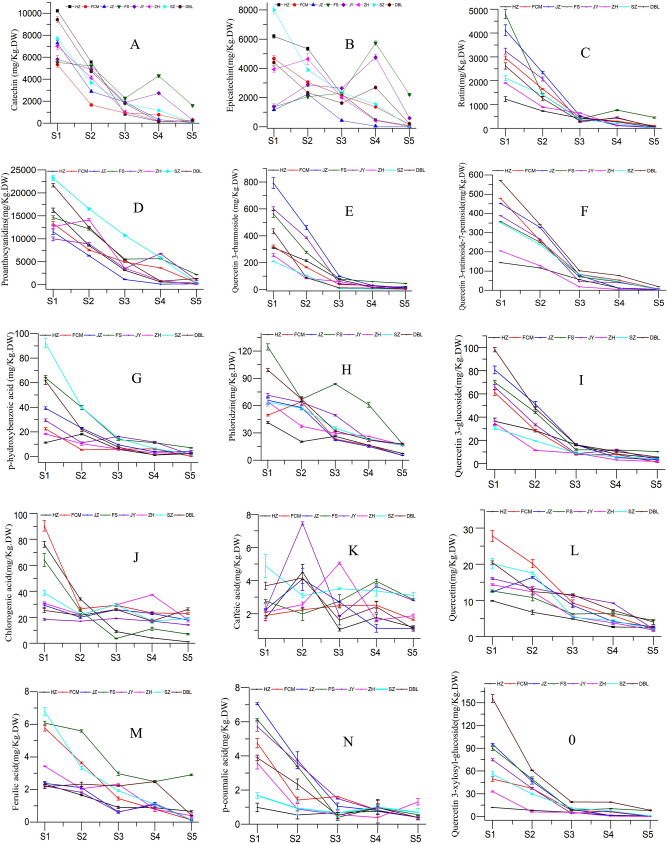
Change in the phenolic profile of jujube fruit in various developmental stages.

Phenolic compounds have health benefits because they are antioxidants. Their antioxidant properties may be due to scavenging radicals via electron transfer mechanisms and chelating with transition metals that are involved in generating free radicals^4^. In the present study, fifteen phenolic compounds were quantified, including hydroxybenzoic acid (p-hydroxybenzoic acid), hydroxycinnamic acids (caffeic acid, p-coumaric acid, and ferulic acid), flavonoids (rutin, quercetin, phloridzin, quercetin-3-glucoside, quercetin-3-rhamnoside, quercetin 3-xylosyl-glucoside, and quercetin 3-rutinoside-7-pentos) and other phenolic acids (chlorogenic acid) and proanthocyanidins (catechin, epicatechin, and proanthocyanidins) using HPLC–DAD analysis (Fig. 8). Among these proanthocyanidins, catechin, epicatechin and rutin were found to be the predominant forms (Fig. 9, Supplementary Table 1). All individual compounds were found to be significantly different (*P* < 0.05) from the total phenolic compounds in all cultivars in various developmental stages. The concentration of individual phenolic compounds was found to decrease with increasing fruit maturity. The maximum phenolic compound content was found in the SZ cultivar (41.00 g/kg DW), followed by the JY cultivar (20.41 g/kg DW) in the green stage (S1), while the lowest content was recorded in the ZH cultivar (0.28 g/kg DW), followed by the FS cultivar (6.49 g/kg DW) in the fully mature stage (S5). This finding was consistent with those of studies reported by Wang^36^. Phenolic compounds, such as procyanidins, catechin, and epicatechin, were found to be predominant in the young fruit stage (S1); these compounds are directly linked with sensory attributes such as bitterness and astringency^37^. These unpleasant flavor characteristics prevent their consumption. However, these unpleasant taste characteristics were reduced as the phenolic concentration decreased during ripening, resulting in a pleasant and sweet flavor in jujube fruit.

### Antioxidant activity (AOX)

**Figure 10:**
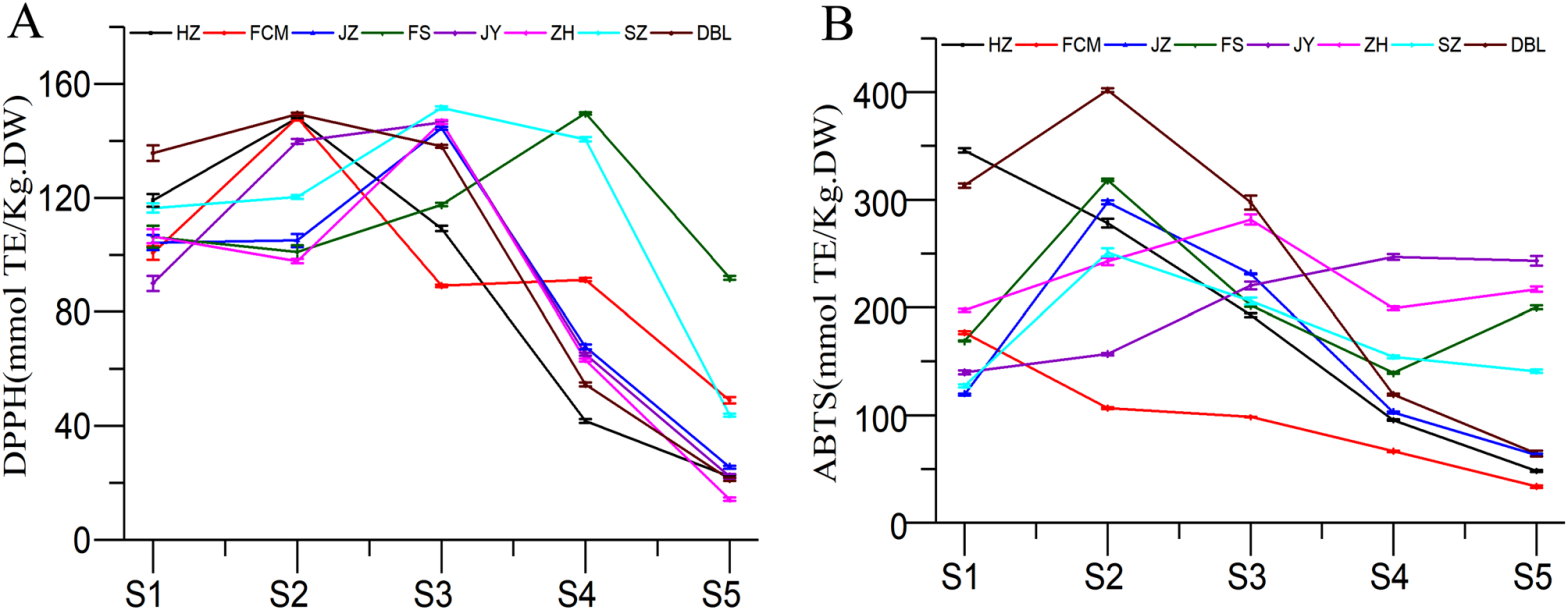
Antioxidant activity shown by (**A**) DPPH and (**B**) ABTS methods in extracts of jujube fruit.

Determination of antioxidant activity is very important because it provides information about the health benefits and functional quality of food when the antioxidant compound is unknown^38^. The antioxidant activity of jujube fruit was determined in terms of free radical scavenging capacity by using DPPH and ABTS analysis. The antioxidant activity of the jujube fruit was obtained in terms of its free radical scavenging capacity, as shown in Fig. 10. In the present investigation, the antioxidant activity in the jujube fruit was found to be significantly different (*P* < 0.05) in the different developmental stages. The DPPH radical scavenging activity of jujube fruit took place from S2 to S4 (Fig. 10A), where they reached the highest value, which then decreased with increasing fruit maturity. The highest peak was obtained in the cultivars of SZ, with a value of 151.54 mmol Trolox/kg DW (S3). Although the highest peak of FS (149.64 mmol Trolox/kg DW, S4) is slightly lower than that of SZ, the DPPH value of FS (91.95 mmol Trolox/kg DW) was the highest among all cultivars in stage 5. The results suggest that the latter reached the highest peak of jujube fruit, and a higher DPPH value could be obtained in the final development stage. For the antioxidant activity studied in ABTS, something similar to DPPH occurs, which shows its maximum activity in all the cultivars from S1 to S4 (Fig. 10B) and decreases considerably as the fruit ripens. The highest value was obtained in the cultivar ‘DBL’, with values of 401.94 mmol Trolox/kg DW (S2). Similar to the DPPH change of FS, JY showed the highest ABTS value (243.30 mmol Trolox/kg DW) among all cultivar in stage 5, but its highest peak was obtained in stage 4. Overall, the highest DPPH value was not only found in FS, but a relatively high ABTS value was also found in FS. This suggests that FS might have an excellent effect on scavenging free radicals. In the present study, antioxidant activity was found to decrease with increasing maturity, which was similar to the findings of studies reported by other researchers^21,39^. The varying antioxidant capacity of fruit might be due to variations in the cultivar, soil conditions, postharvest practices, and maturity of the fruit^20^.

### Principal component analysis (PCA)

Principal component analysis (PCA) is a well-known statistical technique was used to understand the relationships among various quality attributes of the different jujube cultivars. Wand^40^ reported that this technique was used to categorize the cultivars into subgroups in an effort to explore the existing differences among the groups. The results obtained from all chemical analyses were used as variables to anticipate resemblance and difference in a PCA where the dimensionality of numerical datasets was reduced^41^. Generally, the principal components (PCs) have more than 85% cumulative reliability of the original dataset, and then these PCs can replace the original one^41^.

**Figure 11:**
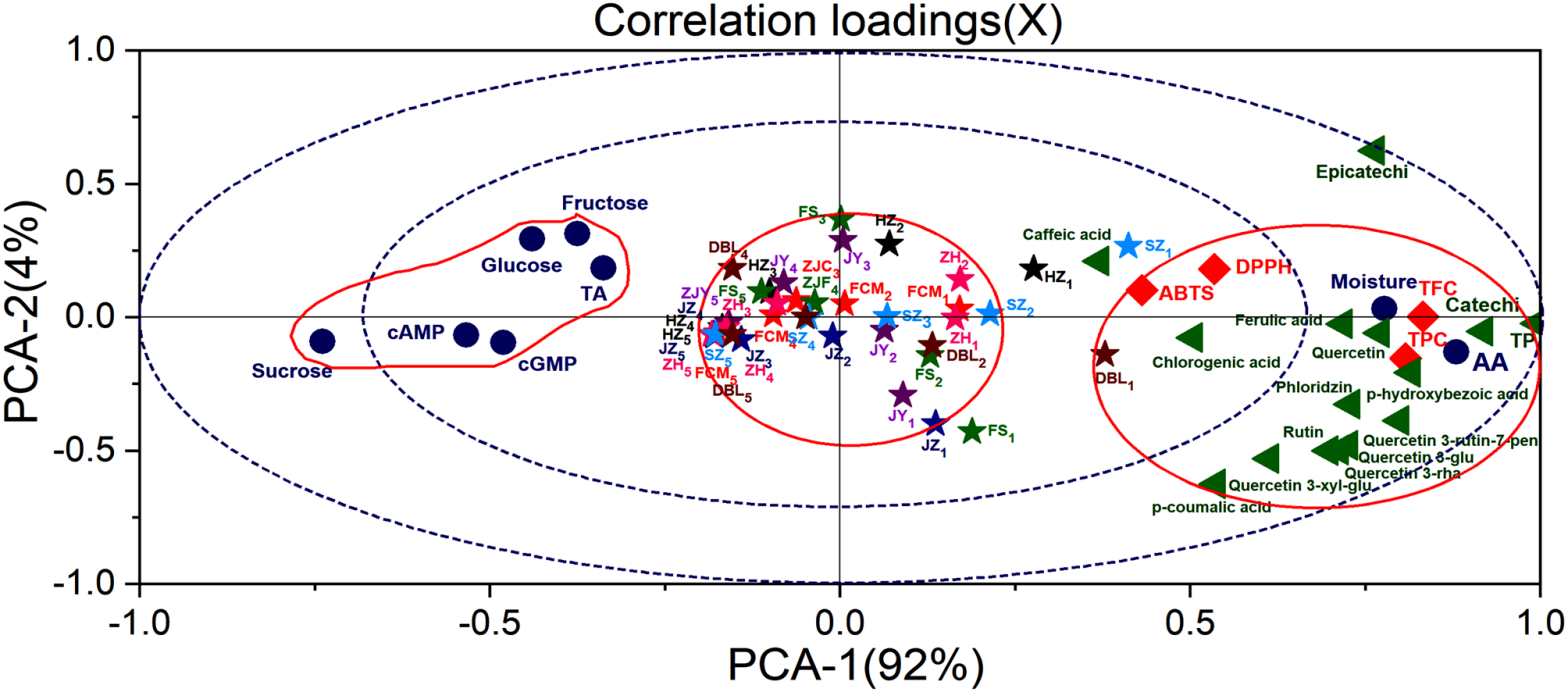
Correlation loading plots of principal component analysis (PCA).

In detail, different cultivars and maturity indices were appropriately divided into three groups (Fig. 11). The first group (PC1), second group (PC2) and third group (PC3) accounted for approximately 92, 4 and 2%, respectively, of the total variability, as indicated in Fig. 11. Moisture, AA, TPC, TFC, phenolic compounds, and antioxidant activities constituted the first (PC1) and second groups (PC2). The third group (PC3) mainly consisted of TA, fructose, glucose, sucrose, cAMP, and cGMP of the cultivars scattered on the left plane (Fig. 11). The correlation loading plot demonstrated that group one had high levels of these indices during the early developmental stage, while group three had high levels of these indices in the final developmental stage of all jujube cultivars. AA, TPC, TFC, phenolic compounds, and antioxidant activities are characteristic components of unripe jujube fruit, while fructose, glucose, sucrose, cAMP, and cGMP are characteristic components of mature jujube fruit according to PCA of the cultivars and indices of the cultivars.

## Conclusion

In conclusion, eight jujube cultivars harvested from Xinjiang Province were differentiated on the basis of physicochemical properties and antioxidant activity. This finding determines the statistical significance in all developmental stages of the different cultivars. The positive/negative correlations of physicochemical properties of the jujube cultivars were validated by correlation analysis. Principal component analysis indicated that maturity stages affected the accumulation of these parameters in all cultivars of jujube fruits. The green fruit (S1) contained the highest level of phenolic compounds and antioxidant activity, whereas the red maturity stage (S5) was rich in sugars, cAMP, and cGMP. According to the findings, the accumulation of sugars in jujube fruits occurs mainly in the maturity stage (S5); thus, fruits in the maturity stage can be used to prepare processed products such as powder, juice, and wine. However, the S1 stage was the source of natural antioxidants in the diet. Among the eight cultivars studied, the highest levels of fructose, fructose and sucrose were found in FCM, FS and JZ, respectively, but the highest content of total sugars was found in HZ, which is a good source of carbohydrates; the highest content of cAMP and cGMP were found in JY, which could be used as potential healthcare foods; and the highest levels of ascorbic acid, catechin, epicatechin, rutin, proanthocyanidin and antioxidant activity were found in ‘FS’, which could be used as potential natural antioxidants. These findings assist in determining the postharvest quality for harvesting, screening, sorting, marketing and processing. Moreover, the mechanisms responsible for the variation in gene expression and enzyme activity remain unclear and need to be investigated through further research.

## Supplementary Information

Supplementary Table 1.
