# Supplementary material for: Physicochemical and antioxidant activity of fruit harvested from eight jujube (Ziziphus jujuba Mill.) cultivars at different development stages
Source: Sci Rep. 2022 Feb 10;12:2272. doi: 10.1038/s41598-022-06313-5 (PMC8831640; doi:10.1038/s41598-022-06313-5)
Supplement: Supplementary file 1 — Supplementary Table 1. [file 41598_2022_6313_MOESM1_ESM.docx]

Supplemented table 1 Change in phenolic profile of jujube fruit at various development stages

| cultivar | Total proanthocyanidin | | | | | Catechin | | | | |
| --- | --- | --- | --- | --- | --- | --- | --- | --- | --- | --- |
|  | S1 | S2 | S3 | S4 | S5 | S1 | S2 | S3 | S4 | S5 |
| HZ | 16203.58±478.91c | 8533.41±155.08i | 3133.80±78.70m | 621.00±40.96p | 324.45 ±14.41 | 10220.66±126.81a | 5560.49±118.72c | 807.74±26.40k | 153.70±7.60 l | 174.59±5.53l |
| FCM | 13320.57±424.43de | 7555.84±183.43j | 4926.94±67.30l | 3652.85±59.60m | 430.27 ±9.35p | 5320.12±223.73d | 1659.93±117.39j | 931.65±35.51k | 766.44±28.46 k | 82.23±6.70 l |
| JZ | 11534.26±613.45f | 6308.60±108.63k | 1131.66±65.33o | 134.02 ±2.08q | 185.12 ±5.74q | 7281.06±220.60b | 2889.44±134.02h | 1916.89±56.86j | 320.70±43.31 l | 55.92±2.20l |
| FS | 14630.86±384.10d | 12160.57±278.70e | 5574.30±64.77l | 5675.37±87.32 l | 2210.40±33.34n | 5531.43±202.50c | 5208.84±118.96d | 2269.95±126.40i | 4304.12±142.18f | 1616.82±75.76j |
| JY | 9980.51 ±420.59h | 8879.14±322.06i | 3546.10±79.64m | 6743.35±89.67k | 981.85 ±37.39op | 5811.66±325.77c | 4904.68±122.73d | 1885.99± 62.72j | 2723.01±82.25h | 290.92 ±6.31l |
| ZH | 12615.61±433.33e | 14147.37±238.99d | 3583.85±38.21m | 888.41± 33.77p | 57.73 ±2.63q | 6997.07± 97.84b | 4132.86±201.84f | 1039.33 ±76.99 | 180.44± 3.81l | 153.70± 2.38l |
| SZ | 23220.95±530.71^a^ | 16508.97±201.32c | 10731.17±131.51g | 5855.94±33.77l | 325.77±2.63p | 7674.98±201.47b | 3681.48±132.62g | 1799.29±82.23j | 1157.12±23.24k | 49.09±3.21l |
| DBL | 21676.71±371.80b | 12503.04±216.13e | 5331.80±78.27l | 760.97±28.72p | 1338.82±33.89o | 9424.03±241.33a | 4718.59±129.06e | 1791.92±19.49j | 142.20±1.90 l | 272.81±2.66l |
| cultivar | Epicatechin | | | | | Rutin | | | | |
|  | S1 | S2 | S3 | S4 | S5 | S1 | S2 | S3 | S4 | S5 |
| HZ | 6193.61±126.81b | 5337.42±118.72c | 2218.76±26.40g | 460.46±7.60i | 77.31±1.53j | 1224.67±103.45i | 723.85±17.93j | 423.73±7.07k | 272.68±5.99l | 50.87±0.97n |
| FCM | 4670.56±223.73d | 3041.82±117.39f | 2138.47±50.51g | 1365.59±20.46h | 160.05±6.70j | 2950.93±81.14d | 1646.35±25.72h | 448.63± 31.65k | 296.68± 8.19l | 111.21± 1.59n |
| JZ | 1160.14±20.60h | 2235.88±74.02g | 425.68±16.86i | 46.13±73.31j | 19.89±0.20j | 4118.03±230.74b | 2338.35±52.20f | 520.17±19.32k | 111.21±3.09n | 28.45±0.75o |
| FS | 1361.80±202.50h | 2061.38±118.96g | 2295.48±26.50g | 5735.40±142.18c | 2210.40±75.76g | 4794.89±188.80a | 1400.80±76.84h | 392.77±12.15k | 763.55±26.50j | 452.31±27.85k |
| JY | 1381.18±65.77h | 2875.61±122.73f | 2640.53±32.72f | 4757.19±182.25d | 591.41±6.31i | 3242.30±122.92c | 2068.88±82.87g | 259.58±15.74l | 455.06±29.92k | 50.63±1.52n |
| ZH | 3933.55±203.24e | 4633.63±376.21d | 1995.03±84.28g | 425.68±7.04i | 19.87±1.45j | 1900.17±17.03g | 888.72±22.27j | 643.62±13.45jk | 127.92±6.93n | 45.96±1.07n |
| SZ | 7996.89±229.06a | 3893.56±109.49e | 2347.42±92.66g | 1540.21±35.90h | 54.20±2.87j | 2111.20±103.41g | 1400.80±53.13h | 377.59±32.88kl | 202.53±7.97m | 30.93±1.69no |
| DBL | 4403.26±188.07d | 2333.37±50.60g | 1625.26±35.65h | 2687.73±39.68f | 216.51±5.29ij | 2616.78±132.88e | 1252.33±95.48hi | 304.25±8.85l | 427.55±9.59k | 73.65±1.14n |
| cultivar | p-Hydroxybenzoic acid | | | | | Chlorogenic acid | | | | |
|  | S1 | S2 | S3 | S4 | S5 | S1 | S2 | S3 | S4 | S5 |
| HZ | 11.29±0.50h | 18.21±0.71f | 5.76±0.59j | 1.31±0.12m | 2.82±0.42l | 76.15±2.22b | 34.05±0.84de | 8.96±0.82k | 4.06±0.10l | 1.13±0.01m |
| FCM | 22.45±0.98e | 5.32±0.25 | 5.99±1.00j | 6.16±0.57j | 0.10±0.11n | 90.34±3.99a | 26.54±1.21f | 29.30±1.15e | 23.35±0.98g | 22.90±0.63g |
| JZ | 39.24±1.09c | 22.94±0.78e | 9.49±0.46 | 3.77±0.59k | 3.94±0.77k | 29.24±1.62e | 20.00±1.01g | 25.84±0.79f | 22.90±0.86 | 17.93±0.14h |
| FS | 64.00±1.93b | 39.77±1.68c | 13.69±0.84g | 11.13±0.61h | 7.05±0.26i | 64.00±5.05c | 25.14±0.82f | 3.69±0.35l | 11.13±1.27j | 7.05±0.60k |
| JY | 29.34±1.16d | 11.07± 0.80h | 16.14±0.35f | 11.71±0.67h | 1.34±0.08m | 18.43±0.77h | 16.96±0.10h | 19.04±0.19h | 17.00±0.18h | 14.15±0.22i |
| ZH | 18.33± 0.58f | 10.39±1.33h | 6.02±0.75j | 2.78±0.41l | 2.74±0.87l | 30.77±1.08e | 21.99±0.96g | 29.52±0.72e | 37.25±0.33d | 17.98±1.56h |
| SZ | 92.29±3.85a | 39.77±1.24c | 14.67±0.37g | 6.46±0.44ij | 1.59±0.13m | 38.61±1.86d | 22.05±0.86g | 29.53±0.76e | 17.00±1.87h | 19.01±0.86h |
| DBL | 61.50±2.97b | 21.66±1.50e | 7.54±0.66i | 1.08±0.47m | 2.27±0.39l | 25.30±1.56f | 21.41±1.11g | 25.95±0.77h | 17.07±1.05h | 26.11±1.13f |
| cultivar | Caffeic acid | | | | | p-Coumalic acid | | | | |
|  | S1 | S2 | S3 | S4 | S5 | S1 | S2 | S3 | S4 | S5 |
| HZ | 1.73±0.14h | 4.52±0.46b | 1.04±0.09i | 1.77±0.21h | 1.18±0.09i | 0.98±0.25g | 0.54±0.24i | 0.68±0h | 0.75±0.69h | 0.40±0j |
| FCM | 1.89±0.18h | 2.24±0.07g | 2.48±0.07g | 2.51±0.21g | 1.64±0.04h | 4.75±0.2c | 1.41±0.17f | 1.62±0f | 0.81±0.40h | 0.39±0j |
| JZ | 2.24±0.08g | 4.08±0.67c | 2.71±0.03f | 1.12±0.24i | 1.10±0.15i | 7.06±0.06a | 3.75±0.50d | 1.05±0.18g | 0.80±0.03h | 0.39±0.14j |
| FS | 2.77±0.09f | 1.98±0.39h | 2.75±0.40f | 3.96±0.12c | 2.86±0.07f | 6.13±0.06b | 3.38±0d | 0.39±0.17j | 0.96±0.16 | 0.56±0i |
| JY | 2.24±0.02g | 7.45±0.09a | 1.85±0.02h | 3.66±0.08d | 2.81±0.01f | 5.72±0.27b | 3.58±0.17d | 1.49±0.02f | 0.81±0.21h | 0.40±0.04j |
| ZH | 2.09±0.52g | 2.55±0.15g | 5.05±0.06b | 1.61±0.10h | 1.88±0.09h | 3.58±0.34d | 0.88±0.33g | 0.56±0.26i | 0.39±0.58j | 1.31±0.19f |
| SZ | 4.88±0.70b | 3.09±0.13e | 3.51±0.05d | 3.37±0.31e | 3.11±0.17e | 1.68±0.17f | 0.93±0.39g | 0.65±0.24h | 0.99±0.17g | 0.69±0.23h |
| DBL | 3.69±0.21d | 4.16±0.32c | 1.61±0.2h9 | 2.42±0.33g | 1.09±0.07i | 3.88±0.16d | 2.34±0.30e | 0.52±0.19i | 0.97±0.42g | 0.39±0.13j |
| cultivar | Ferulic acid | | | | | Phloridzin | | | | |
|  | S1 | S2 | S3 | S4 | S5 | S1 | S2 | S3 | S4 | S5 |
| HZ | 2.32±0.16f | 1.66±0.05g | 0.91±0.07i | 0.88±0.03i | 0.66±0.04k | 41.38±1.23i | 20.40±0.68n | 26.31±0.70m | 16.48±0.20op | 7.41±0.10q |
| FCM | 5.76±0.18c | 3.64±0.05d | 1.45±0.09h | 0.79±0.14j | 0.13±0.04m | 49.51±0.91h | 64.33±3.70f | 23.07±0.27n | 16.39±0.68op | 5.38±0.03q |
| JZ | 2.38±0.04f | 2.12±0.07f | 0.62±0.08k | 1.08±0.13i | 0.14±0.06m | 65.95±3.20ef | 57.92±1.73g | 22.41±0.98n | 15.10±0.94p | 5.38±0.31q |
| FS | 6.08±0.11b | 5.59±0.07c | 2.97±0.11e | 2.48±0.04f | 2.90±0.05e | 124.54±3.44a | 67.03±2.06e | 83.79±0.70c | 60.98±2.75f | 17.90±0.80o |
| JY | 2.21±0.06f | 1.82±0.03g | 0.61±0.03k | 1.14±0.04i | 0.14±0.21m | 71.49±1.84d | 63.64±2.70f | 49.51±0.86h | 22.28±1.69n | 16.66±0.92o |
| ZH | 3.43±0.03d | 2.05±0.04f | 2.33±0.02f | 0.80±0.13j | 0.39±0.11l | 64.33±3.22f | 37.30±1.45j | 29.73±1.25l | 26.31±0.18m | 16.74±0.38o |
| SZ | 6.78±0.25a | 3.32±0.10d | 1.92±0.06g | 1.09±0.07i | 0.14±0.09m | 63.80±4.35f | 57.30±2.08g | 35.26±1.54j | 23.36±0.44n | 16.66±0.74o |
| DBL | 2.18±0.13f | 2.32±0.12f | 2.22±0.02f | 2.48±0.07f | 0.41±0.02l | 99.16±1.84b | 67.03±2.70e | 32.33±0.86k | 22.41±1.69n | 17.90±0.92o |
| cultivar | Quercetin 3-rhamnoside | | | | | Quercetin | | | | |
|  | S1 | S2 | S3 | S4 | S5 | S1 | S2 | S3 | S4 | S5 |
| HZ | 311.05±5.60g | 214.84±6.49i | 74.62±2.99m | 18.01±0.86q | 21.56±1.53q | 9.86±0.10h | 6.70±0.59i | 4.85±0.08k | 2.64±0.18m | 2.53±0.26m |
| FCM | 322.11±9.35g | 166.08±3.35j | 37.48±1.34 | 32.20±2.16p | 2.69±0.38t | 27.80±1.54a | 20.14±1.12b | 9.25±0.88h | 5.73±0.08j | 2.08±0.14n |
| JZ | 792.34±39.58a | 460.67±15.16d | 98.07±3.03k | 30.74±1.24p | 14.30±0.83r | 12.36±0.10f | 16.36±0.24d | 8.60±0.64h | 4.12±0.42kl | 2.67±0.23m |
| FS | 563.29±26.17c | 277.65±7.86h | 78.58±3.19m | 60.18±0.22n | 46.23±1.33o | 12.58±0.30f | 10.79±0.95g | 6.23±0.08ij | 6.39±0.36i | 4.51±0.15k |
| JY | 600.70±15.02b | 385.60±5.83f | 43.54±2.70o | 28.91±0.45p | 9.76±0.95s | 16.00±0.42d | 13.39±0.31g | 11.23±0.39g | 9.25±0.07h | 2.08±0.45n |
| ZH | 256.08±11.04h | 83.28±3.13l | 59.76±2.62n | 16.66±0.15q | 4.15±0.83t | 14.33±0.25e | 12.36±0.96fg | 5.41±0.27j | 3.76±0.47l | 1.79±0.41n |
| SZ | 210.14±1.41i | 102.61±5.45k | 8.49±0.71s | 4.69±0.47t | 3.27±0.06t | 20.13±1.44b | 17.54±0.35c | 5.28±0.21j | 4.41±0.23k | 1.96±0.14n |
| DBL | 433.47±18.45e | 88.71±3.37km | 13.13±0.22 | 11.55±0.69s | 4.09±0.35t | 20.44±0.44b | 12.41±0.63fg | 11.52±0.26g | 7.23±0.19i | 4.00±0.69kl |
| cultivar | Quercetin 3-glucoside | | | | | Quercetin 3-xylosyl-glucoside | | | | |
|  | S1 | S2 | S3 | S4 | S5 | S1 | S2 | S3 | S4 | S5 |
| HZ | 36.47±2.78g | 28.43±1.16h | 16.23±0.92j | 8.11±0.17l | 4.97±0.17m | 11.97±0.04l | 8.15±0.27n | 5.47±0.62p | 1.73±0.10q | 0.70±0.06r |
| FCM | 61.14±2.48d | 28.53±0.97h | 7.79±0.36l | 7.89±0.06l | 1.18±0.15o | 49.46±3.26g | 37.08±1.27h | 8.11±0.52n | 5.99±0.06p | 0.00r |
| JZ | 80.68±3.30b | 51.21±2.22e | 16.21±0.30j | 5.17±0.29m | 3.30±0.01n | 95.23±1.20b | 46.46±3.74g | 9.50±0.08m | 1.33±0.06q | 0.36±0.03r |
| FS | 69.99±1.77c | 44.05±0.92f | 11.87±0.95k | 11.76±0.72k | 10.36±0.09k | 89.68±2.69c | 49.35±2.82g | 8.39±0.62n | 10.33±0.43l | 7.70±0.03n |
| JY | 66.05±2.13c | 33.31±0.81g | 8.59±1.15l | 11.72±0.69k | 3.36±0.89n | 74.76±1.33d | 37.08±1.24h | 4.19±0.09q | 6.91±0.24o | 0.00r |
| ZH | 34.76±1.93g | 11.48±0.32k | 9.04±0.31l | 3.17±0.14n | 1.73±0.66o | 32.93±0.81i | 5.90±0.29p | 5.43±0.32p | 0.50±0.04r | 0.00r |
| SZ | 30.50±1.23h | 19.57±0.22i | 8.93±0.28l | 5.80± 0.41m | 4.30±0.45m | 55.79±3.40f | 29.82±1.06j | 11.26±0.12l | 7.27±0.08no | 0.78±0.05r |
| DBL | 98.08±1.90a | 47.43±1.25e | 16.23±0.04j | 10.36±0.80k | 5.67±0.06m | 155.51±5.24a | 60.94±0.50e | 19.04±0.81k | 18.89±0.29k | 8.37±0.32n |
| cultivar | Quercetin 3-rutinoside-7-pentos | | | | |  | | | | |
|  | S1 | S2 | S3 | S4 | S5 |  |  |  |  |  |
| HZ | 143.89±5.28j | 116.52±1.75k | 55.31±2.72n | 11.50±0.57op | 4.44±0.14p |  |  |  |  |  |
| FCM | 478.45±12.92b | 258.52±7.37g | 82.49±2.90m | 52.75±1.09n | 5.05±0.04p |  |  |  |  |  |
| JZ | 452.10±9.90c | 327.23±8.09f | 76.44±2.92m | 10.75± 0.32op | 2.61±0.86p |  |  |  |  |  |
| FS | 357.54±3.90e | 249.87±5.55g | 70.41±4.72m | 42.77±1.42n | 4.08±0.07p |  |  |  |  |  |
| JY | 388.16±3.37d | 265.85±4.40g | 45.03±1.34n | 40.14±0.43n | 7.42±0.33p |  |  |  |  |  |
| ZH | 204.81±8.21i | 128.16±6.39k | 17.84±0.34o | 4.27±0.32ip | 1.84±0.07p |  |  |  |  |  |
| SZ | 351.88±5.51e | 237.30±6.58h | 81.33±0.27m | 45.03± 0.80n | 4.08±0.07p |  |  |  |  |  |
| DBL | 570.67±13.06a | 342.29±10.29e | 101.51±1.02l | 76.66±2.61m | 18.98±0.91o |  |  |  |  |  |

The different lowercase letters in the same column showed significant difference at P<0.05 level.
